# Can a prostate biopsy be safely deferred on PI-RADS 1,2 or 3 lesions seen on pre-biopsy mp-MRI?

**DOI:** 10.1080/2090598X.2022.2119711

**Published:** 2022-09-13

**Authors:** Rickaz Abdul Raheem, Ahsen Razzaq, Victoria Beraud, Richard Menzies-Wilson, Rakan Odeh, Imoh Ibiok, Prashant Mulawkar, Henry Andrews, Iqbal Anjum, Khaled Hosny, Tom Leslie

**Affiliations:** a Speciality Doctor in Urology, Milton Keynes University Hospital, UK; b University of Buckingham Medical School; c Urology Specialist Registrar, Oxford University Hospitals; d Department of Urology, Tirthankar Superspeciality Hospital, Akola, India; e Consultant Urologist, Milton Keynes University Hospital, UK; f Consultant Urological Surgeon, Milton Keynes University Hospital, UK; g Consultant Urological surgeon, St. Helens and Knowsley NHS trust, UK

**Keywords:** prostate cancer, mpMRI scan, PIRADS, active surveillance

## Abstract

**Introduction:**

Multi-parametric magnetic resonance imaging (mp-MRI) is currently used to triage patients with suspected prostate cancer, before deciding on prostate biopsies. In our study, we evaluated normal and equivocal pre-biopsy mp-MRIs to see whether it is safe to avoid biopsy with such findings.

**Methods:**

A retrospective study was conducted at a district general hospital in the UK between August 2017 and July 2018. Patients with negative and equivocal prebiopsy mp-MRI with high clinical suspicion of cancer had proceeded to biopsy. MRI reports with prostate imaging reporting and data system (PI-RADS) scores 1, 2, 3 and normal MRI were evaluated against the transrectal ultrasound-guided prostate biopsy (TRUS-PB) outcomes to demonstrate benign pathology, clinically insignificant or clinically significant cancer (csCa). CsCa was defined as Gleason score (GS) ≥3 + 4.

**Results:**

Out of 265 mp-MRIs studied, five (1.9%) were PI-RADS 1, 109 (41.1%) and 84 (31.7%) were PI-RADS 2 and 3 lesions respectively; 67 (25.3%) were reported as normal. Seventy-five (27.3%) patients did not have biopsies following their MRI and 73.3% (51/75) of them had benign feeling prostate. Negative MRIs (PI-RADS 1, 2 and normal MRI) showed 8.8% and PI-RADS 3 lesions demonstrated 11.9% csCa. Negative predictive value for normal MRI was 91.2%. Mean PSA density (PSAD) among the benign, GS 3 + 3 and csCa was 0.14, 0.16 and 0.27 ng/ml/ml respectively and this was statistically significant (*p* < 0.001). The average percentage of cancer found in GS 3 + 3 and csCa was 3.2% and 20.1%, respectively.

**Conclusion:**

Avoiding TRUS-PB following normal or equivocal mp-MRI should carefully be decided as 18.5% of cancer was demonstrated in this group and 9.8% of those who were diagnosed with cancer were csCa. PSAD and DRE findings provide additional information to help with this decision.

## Introduction

Prostate cancer is diagnosed in one in six men during their lifetime [1]. Various studies have shown over-diagnosis and over-treatment of prostate cancer [2,3]. In the UK, suspected prostate cancer patients are referred through a fast track two-week wait pathway based on an elevated age-specific prostate-specific antigen (PSA) or abnormal prostate on digital rectal examination (DRE) [4].

Traditionally, prostate cancer was diagnosed with trans-rectal ultrasound (TRUS) guided prostate biopsies under local anaesthesia. However, in the recent past prostate MRI (magnetic resonance imaging) scans have yielded more diagnostic value in prostate cancer and increasing numbers of men are subjected to these scans [5]. The European Society of Urogenital Radiology (ESUR) has recommended the use of multi-parametric MRI (mp-MRI), including T1 and T2-weighted imaging, diffusion-weighted imaging (DWI) and dynamic contrast-enhanced (DCE) imaging for the diagnosis of prostate cancer [1,3,6], and this is reported according to a five-point Prostate Imaging Reporting and Data System (PI-RADS) probability scale, higher the score the most likely to have cancer [1] .

Several studies were carried out to validate the presence of clinically significant prostate cancer based on PI-RADS score and most have concentrated on PI-RARDS 3,4 and 5 lesions [7,(8,9]. Some looked at the feasibility of safely avoiding a prostate biopsy following a pre-biopsy mp-MRI. For example, the landmark PROMIS study claims pre-biopsy mp-MRI can avoid a primary biopsy in 27% of the patients [10]. In addition, the PRECISION study demonstrated a better detection of clinically significant cancer in the MRI targeted biopsy group than the standard prostate biopsy group (38% vs 26%) [7]. Hence, clinicians are only considering performing targeted biopsies and avoiding systematic biopsies. However, a decision not to perform a biopsy on the normal areas on mp-MRI scan must be decided carefully as there is still a possibility that clinically significant cancer could be found in those areas. A study from the USA reported 16% clinically significant cancer could be missed if systematic biopsies were avoided based on a non-suspicious target on MRI [11]. Another UK based study has shown low sensitivity and specificity for mp-MRI to avoid a trans-rectal prostate biopsy [12]. The latest NICE guidelines also state 11–28% of people with low-risk MRI actually have clinically significant cancer [13]. Therefore, validation of mp-MRI scans against the biopsy outcome in normal. MRI, lower and equivocal PI-RADS scores will help in decision making when managing patients with suspected prostate cancer.

Our objectives were to study the outcome of biopsies done in normal as well as PI-RADS 1, 2 and 3 lesions on mp-MRI in clinically suspected prostate cancer, and to measure the percentage of clinically significant prostate cancer among them. There is scarce evidence available to compare the biopsy outcome for reported PI-RADS 1 and 2 lesions found on mp-MRI [14]. In clinical practice, most men inquire about prostate MRI for prostate cancer diagnosis and are also keen to avoid prostate biopsies if the MRI showed normal findings. We looked at the outcome of these findings, along with the equivocal PI-RADS 3 lesions in the pre-biopsy mp-MRIs done at a UK based district General Hospital, to see whether it is safe to defer a prostate biopsy when the MRI shows normal or equivocal findings and to examine the factors which influence the decision to avoid a biopsy in such clinical situation.

## Methods

### Study design

A retrospective cohort study was conducted by reviewing the reports of mp-MRI prostate scans done in biopsy naïve patients at Milton Keynes University Hospital (MKUH) from 1/8/2017 to 31/7/2018. Scans that were reported as PI-RADS 1, 2 or 3 lesions were identified and included in the study, as well as those reported as ‘normal MRI’ or ‘no abnormalities detected’. All the MRIs were performed using a 1.5 T scanner and PI-RADS v2 was used for reporting. All these mp-MRI scans were reported by experienced radiologists. Transrectal ultrasound-guided prostate biopsies were performed by consultant urologists and an experienced associate specialist in urology. Cognitive fusion has been used if a target was identified. Pathology specimens had been reported by experienced consultant pathologists. All the MRI scans and histology reports were discussed in the Urology cancer MDT in the same hospital.

MRI scans which were reported as PI-RADS 4 or 5 lesions, ‘highly suspicious for cancer’, ‘likely to have cancer’ or which showed definitive evidence of cancer such as extra-prostatic involvement, lymph nodes or bony metastasis had been excluded. In addition, patients who had follow up MRI; either as part of active surveillance protocol or those who were previously diagnosed with prostate cancer. Patients who had trans-perineal biopsies were also excluded.

### Data collection and statistical analyses

We extracted our data from the hospital medical records including age, latest PSA before MRI scan, MRI reported prostate volume and the digital rectal examination (DRE) finding of the prostate.

We primarily looked at those who had biopsies; histopathology was divided into benign or malignant. We assessed those with a cancer diagnosis for more details including Gleason score, cancer percentage, the maximum cancer core length, total number of cores and the number of positive cores in the biopsy specimen. Gleason score ≥7 were considered clinically significant. A further comparison was made between clinically significant and insignificant cancer groups. Since the EAU guidelines mention PI-RADS ≤2 as negative MRI [14], all the MRIs which were reported as PI-RADS 1,2 and normal MRI or no abnormalities detected were considered ‘normal/negative MRI’ for analyses.

Secondly, patients who did not undergo TRUS biopsies were studied separately. We looked at significant factors that contributed to decision-making versus those who had prostate biopsies.

Statistical Package for Social Science (IBM SPSS V 20) was deployed to analyse the data and the test results were concluded based on the level of significance α = 0.05, otherwise mentioned. Power calculations were done assuming 15% of PI-RADS 1, 2, 3 or normal mp-MRI has clinically significant cancer. This was estimated based on the NICE guidelines which stated low-risk mp-MRI really has 11–28% of clinically significant cancer [13]. Besides, for PIRADS 3 lesions, PRECISION study has shown 12% clinically significant cancer [7] and another study by Thai et al. has shown 11% [9]. These data also were taken into consideration when the sample size was calculated. When the probability of type I error (α) was set to 0.05 and the probability of type II error (β) at 0.1, at least 106 samples would have guaranteed 90% power. Similarly, a minimum sample size of 150 will give a power percentage of 97.69% (α = 0.05, β = 0.23) [15].

## Results

### Study population

**Figure 1.**
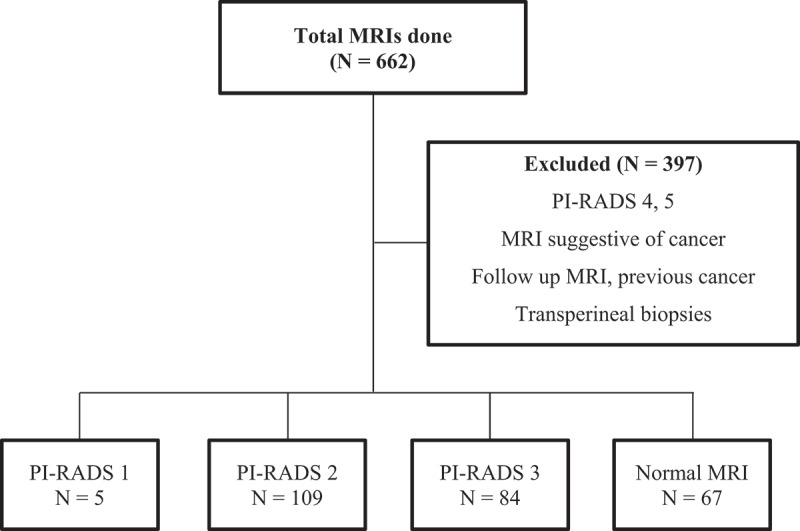
Summary of inclusion and exclusion of the MRIs to the study.

A total of 662 MRI scans had been performed within the study period. 265 of them showed either PIRADS 1, 2 or 3 lesions or were reported as normal. The remaining 397 scans fell within the category of exclusion criteria (Figure 1). Among the 265 scans studied, 5 (1.9%) showed PI-RADS 1 lesion, whereas 109 (41.1%) and 84 (31.7%) showed PI-RADS 2 and 3 lesions respectively. 67 (25.3%) were reported as normal scans. The basic characteristics of the study populations are shown in Table 1. A bivariate Pearson’s correlation coefficient was calculated between age, PSA, prostate volume, and PSA density at 1% of the level of significance. Age showed weak positive correlation with PSA and Prostate volume *r*(265) = 0.219, *p* < 0.001 and *r*(265) = 0.249, *p* < 0.001 respectively. However, there was no significant correlation between age and PSA density.

**Table 1.** Comparison of mean characteristics between the study population, MRI findings, and the biopsy outcome *.

Mean characteristics	Study population (N = 265)	Biopsy done (N = 190)	Biopsy not done (N = 75)	Total cancer (N = 49)	ciCa (N = 23)	csCa (N = 26)
Age (years)	65.6 ± 9.6	64.8 ± 9.1	67.6 ± 10.3	65	63	67
PSA (ng/ml)	9.16 ± 8	9.2 ± 8.6	9.2 ± 6.7	10.9	9.2	12.4
Prostate volume (ml)	71.3 ± 43.7	71.2 ± 44	71.7 ± 43.3	60.2	69.4	52
PSA density (ng/ml/ml)	0.17 ± 0.2	0.16 ± 0.17	0.13 ± 0.08	0.22	0.16	0.27
PI-RADS 1	**5 (1.9%)**	5	0	0	-	-
PI-RADS 2	**109 (41.1%)**	76	33	**21/109 (19.3%)**	11 (10%)	10 (9.2%)
Normal MRI	**67 (25.3%)**	49	18	**8/67(12%)**	2 (3%)	6 (9%)
PI-RADS 3	**84 (31.7%)**	60	24	**20/84 (23.8%)**	10 (11.9%)	10 (11.9%)
Benign DRE	153 (57.7%)	51.6%	73.3%	45%	50%	39%
Suspicious DRE	112 (42.3%)	48.4%	26.7%	55%	50%	61%

*Plus-minus values are means ± SD.

*ciCa – clinically insignificant cancer, csCa – clinically significant cancer*

**Figure 2.**
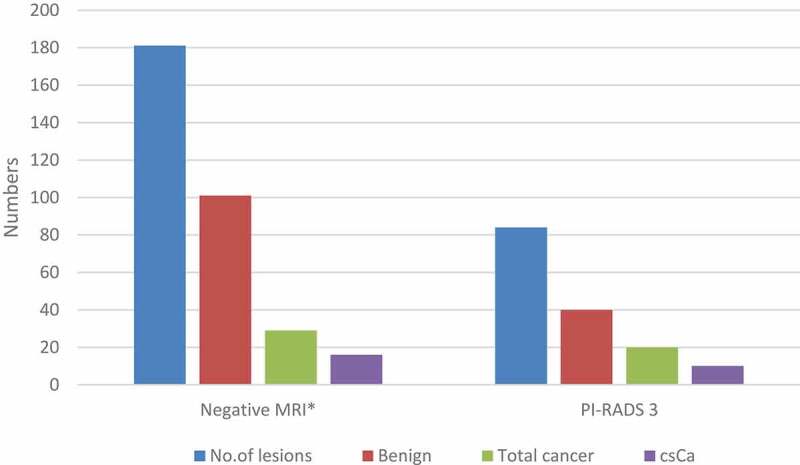
Characteristics of biopsy outcome.

### The outcome against MRI findings

Of the 265 patients whose MRIs were included in the study, 190 (71.7%) have had prostate biopsies following their MRI. 141/190 patients (74.22%) showed benign pathology whereas 49/190 (25.78%) were cancers. Twenty-six of these cancers were clinically significant. None of the five PI-RADS 1 lesions showed cancer. When PI-RADS 1,2 lesions and normal MRI are considered as ‘negative MRI’ (181 total MRIs in this category), this cohort showed 29 cases of cancer (16%) and out of them, 16 cases were clinically significant (8.8%). Number of cancers found with different categories are detailed in Figure 2.

The patients who didn’t undergo biopsy (N = 75) were the ones who were not willing or those with low clinical suspicion for cancer when other clinical parameters were considered. Fifty-one (68%) of them had PI-RADS 2 or normal MRI. Fifty-five (73.3%) of these patients who didn’t have biopsies had benign feeling prostate. Among 84 PI-RADS 3 lesions, 24 of them (44%) avoided biopsy (Table 1). Independent t-tests performed between the groups of ‘biopsy done’ and ‘biopsy not done’ to compare the means of age (p = 0.006) and PSA density (p < 0.0001) were found to be significant. A Chi-squared test was performed to assess the relationship between PI-RADS reading and DRE findings with the cohort of whether biopsy was done or not has shown no significant, $\chi^{2}\left(3,N=265\right)=2.254,p=0.521$. However, the relation between DRE finding with this group was significant, $\chi^{2}$ (1,N = 265) = 10.429, p = 0.001.

### Analysis based on histology

Histopathological diagnoses of these biopsies were evaluated in detail. First, a benign vs a cancer diagnosis was established. Two-thirds of biopsied specimens (141 of 190) showed benign pathology. Benign diagnoses included prostatic hyperplasia, inflammation, high-grade PIN, and atypical small acinar proliferation (Table 2). All the clinically insignificant and significant cancers were studied in detail including Gleason score, percentage of cancer, the maximum cancer core length, site of cancer and the number of positive cores from the total cores. It has been noted that higher volume disease has been noted with clinically significant disease compared to clinically insignificant (Table 3).

**Table 2.** Distribution of diagnosis in biopsied specimens.

Diagnosis	Frequency	Percentage
BPH/inflammation	111	58.4
PIN	28	14.7
ASAP	2	1.1
GS 3 + 3	23	12.1
GS 3 + 4	20	10.5
GS 3 + 5	1	0.5
GS 4 + 3	2	1.1
GS 4 + 5	2	1.1
GS 4 + 4	1	0.5
Total	190	100

*BPH – Benign Prostatic Hyperplasia, PIN – Prostate Intraepithelial Neoplasia, ASAP – Atypical Small Acinar Proliferation, GS- Gleason Score*

**Table 3.** Comparison of biopsy findings between total, clinically insignificant and significant cancer.

Biopsy findings	Total cancer (N = 49)	ciCa (N = 23)	csCa (N = 26)
Percentage Ca	13.8	3.2	20.1
MCCL (mm)	4.5	2.5	6.1
Positive cores	4.2	2.1	6.1
Total cores	16.8	17.5	16.1
% Positive cores to total cores ratio	25	12	37.9

*MCCL – Maximum Cancer Core Length, ciCa – clinically insignificant Cancer, csCa – clinically significant Cancer*

### DRE finding and the diagnosis

DRE findings were studied to find out if the prostate was suspicious or benign on clinical examination. Two-third of the patients who avoided a biopsy had benign feeling prostate and most of the suspicious prostate resulted in clinically significant cancer (Table 1). A Chi-Square tests of independence confirmed the relationship between DRE finding and cancer diagnosis as significant ($\chi^{2}$ (9, N = 265) = 18.314, *p* = 0.032).

### Analysis of diagnosed cancer

49 patients (18.5%) in the total study population showed cancer diagnosis. Twenty-three (8.7%) of them were clinically not significant (Gleason score 3 + 3) whereas the remaining 26 (9.8%) were considered as clinically significant (Table 1). These biopsies were studied in detail and data was gathered on cancer percentage, the maximum cancer core length, number of positive cores out of total cores (Table 3).

One-Way ANOVAs were carried out to compare the mean PSA density, cancer percentage, the maximum cancer core length, positive cores, and total cores that showed significance against the diagnosis of cancer (*p < 0.0001*). However, these variables were not significant with the PI-RADS score. An independent sample t-test showed statistical significance between clinically significant cancer and percentage cancer *(p = 0.002*), maximum cancer core length *(p < 0.0001)* and the number of positive cores *(p < 0.0001)*. Age has not shown any significant correlation with the other variables.

PSA has a moderate positive correlation with percentage cancer *(p < 0.0001*), weak positive correlation with maximum cancer core length *(p = 0.025)* and moderate positive correlation with the number of positive cores *(p < 0.0001)*. PSA density showed a significant moderate positive correlation with percentage cancer, maximum cancer core length and number of positive cores *(p < 0.0001)*. Among the patients who were diagnosed with cancer, a significant relationship was seen between the PI-RADS lesions and the DRE finding ($\chi^{2}$ (2, N = 49) = 6.621, *p* = 0.037).

### Discussion

Despite the new advancements in mp-MRI techniques improving prostate cancer diagnosis and staging to a great extent [16] [17], the sensitivity and specificity of MRI to diagnose significant cancer was found to be at a different range in various studies. While the accuracy of MRI prostate to diagnose cancer was reported to be 44–87%, sensitivity and specificity ranged between 58–96% and 23–87%, respectively [18]. This wide range of sensitivity and specificity pose a challenge in clinical practice.

Although the recent multicentre studies such as PRECISION [7] and PROMIS [10] suggested to biopsy the patients only when the MRI is abnormal or to perform targeted biopsies instead of systematic biopsies, this has led to many debates across the world [18]. A valid argument for this arises as 24% detection of clinically significant cancer was seen in negative MRIs in the PROMIS study [10]. Another study from the US has shown 16% clinically significant cancer detected with systematic biopsies with no suspicious MRI target. This study also found 7% of clinically significant cancer in the entirely non-suspicious areas found on abnormal MRI [11]. Therefore, the normal MRIs must be studied to validate the presence of cancer before a decision is made not to biopsy these areas.

In our study, negative MRI has shown 16% of cancer and 8.5% of them were clinically significant. For PIRADS 3 lesions, 23.8% were showing cancer while 11.9% were clinically significant. These findings are comparable with similar results in the literature. Stabile *et al*. reported that 8% of PIRADS 2 lesions and 15% of PIRADS 3 lesions were clinically significant cancer [19] and Thai *et al*. also found 11.1% of PIRADS 3 lesion was clinically significant cancer [9]. Recently, Sathianathen and colleagues demonstrated 8.9% clinically significant cancer with PI-RADS 3 lesions [20]. All those studies defined clinically significant cancer as Gleason score ≥3 + 4 = 7. We also found normal mp-MRI has a 91.2% negative predictive value in the present study.

Interestingly, the percentage of clinically significant cancer changed greatly based on the definition we apply. In some landmark papers, for example in the PROMIS study, the primary definition for clinically significant cancer was considered as Gleason score 4 + 3 [10]. If this definition is applied to our study, only 1 out of 181 normal MRIs and 4.8% (4 out of 84) of PI-RADS 3 lesions showed clinically significant cancer. However, it is notable that the NICE and EAU guidelines include any prostate cancer of Gleason score 7 and above as clinically significant [13,14] and this is the author’s practice as well.

**Figure 3.**
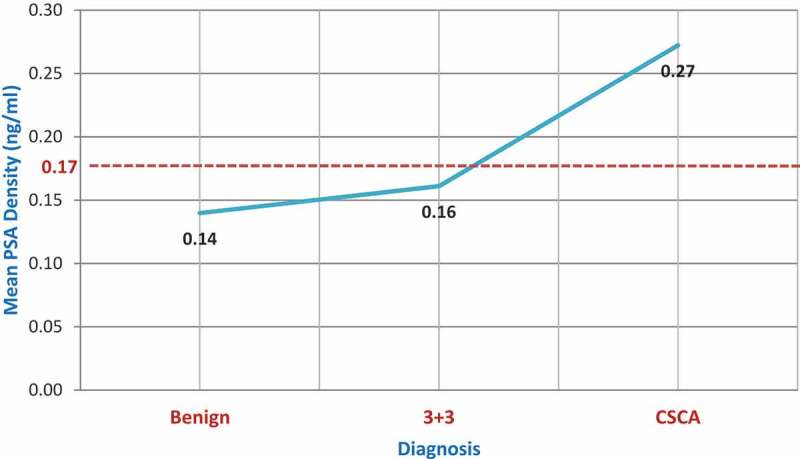
Correlation of PSA density with the biopsy outcome.

We also note that the DRE finding and PSA density (PSAD) could help us to predict significant cancer. A significant relationship has been noted between DRE findings and the final diagnosis. Two-thirds of the patients who had clinically significant cancer had suspicious DRE. Our work also showed that PSA density (PSAD) is a valuable predictor for the diagnosis of cancer. It is evident that an increasing PSAD has a positive influence on the higher grade and high volume of cancer. The mean PSAD among the benign diagnosis was 0.14 ng/ml/ml whereas for Gleason score 3 + 3 = 6 cancer, this was 0.16 ng/ml/ml. However, for the clinically significant cancer group, the mean PSAD was 0.27 ng/ml/ml (Figure 3). PSA density was also positively correlated with other biopsy parameters such as percentage cancer, maximum cancer core length and the number of positive cores. We are aware from previous studies that PSAD of ≥0.30 ng/ml/ml with PIRADS 3 changes are associated with a higher possibility of clinically significant cancer [21]. The present study also showed a mean PSAD of 0.27 ng/ml/ml with PI-RADS 3 lesions when a clinically significant cancer was diagnosed.

Analyses of other characteristics between clinically insignificant (Gleason score 3 + 3) and significant cancers (Gleason score ≥3 + 4) demonstrated important findings. Among the diagnosed cancer cases the average percentage of cancer in the biopsy specimens was 13.8%. On separate analysis, only 3.2% of cancer was found when clinically insignificant cancer was diagnosed whereas, for clinically significant cancer, 20.1% of mean percentage cancer was identified. Similarly, maximum cancer core length was greater when a clinically significant cancer was diagnosed (6.5 mm) compared to clinically insignificant cancer (2.5 mm). Interestingly, a higher percentage of positive cores was noted from the total number of biopsied cores in clinically significant cancer (37.9%) compared to the clinically insignificant group (12%). Therefore, it can be concluded that an increased volume of cancer is harboured with a higher Gleason score. This finding is clinically important in managing patients since the amount of tumour found in the biopsy has been found to influence overall survival [22].

### Is it safe to avoid biopsy in a normal MRI?

Widespread media publicity about prostate cancer and prostate MRI scan has created awareness among men and debates have started on ‘prostate cancer screening’ in the UK in the recent past [23]. In clinical practice, increasing numbers of men are seen with suspected prostate cancer referral that needs further investigations. Prostate MRI is an important tool to detect and localize prostate cancer and aimed to triage and carefully select patients who should undergo biopsy [6]. The present guidelines do not recommend the use of mp-MRI as a screening tool for prostate cancer [20] and also recommend omitting biopsy in PI-RADS 1 or 2 lesions only after explaining the pros and cons to the patient [13].

In this context, knowledge of the possibility of diagnosing cancer in negative or equivocal findings in the MRI surely helps in decision-making to the patient and the clinician as well. In addition to our findings explained in this paper, several other studies have also reported similar figures. Filson *et al*. reported a 16% possibility of missing Gleason score ≥7 cancer if a biopsy was avoided after a normal mp-MRI [24]. Sathianathen *et al*. found 8.8% significant cancer in PI-RADS 3 lesions [20]. Branger *et al*. compared the radical prostatectomy outcome against the preoperative negative mp-MRI and found 60.4% overall unfavourable pathology after surgery and concluded that a negative MRI could not be assured absence of clinically significant cancer [25]. These patients had other parameters to suggest significant disease although the MRI was normal hence finally underwent radical surgery. Therefore, MRI alone might not be the deciding investigation when dealing with patients with suspected prostate cancer. We have noted DRE finding and PSA density are additional factors to consider. Taking these factors into consideration, a detailed discussion with patients should be done before deferring biopsies based on MRI findings. A balanced decision-making could be challenging in this situation between diagnosing significant cancer against over-diagnosing a benign or non-significant cancer which may result in unnecessary over-treatment.

Another aspect to consider in avoiding biopsy based on MRI findings alone is the level of experience of the radiologist who is reporting. It has been a concern that the reporting by radiologists, in general, may not represent the similar accuracy as in the specialized centres in prostate MRIs, therefore, reporting by new radiologists with less experience and training may affect the good clinical practice [26]. One of the limitations in our study is that there was no routine centralized MRI or histopathology review system, however, all these MRI scans and histology reports had been discussed in the departmental Urology cancer MDT meetings.

Avoiding a prostate biopsy purely based on normal MRI is not yet appears advisable. Various other factors also play a role in decision-making in the management plan in men with suspected prostate cancer. Robust research is required to come to such a conclusion regarding non-suspicious MRI and this present study is believed to provide additional value for similar kind of studies to be done in future. Careful consideration is also required when applying research findings from high volume centres to less busy units and regular local audits will ensure quality assuarance in such units.

### Strengths and Limitations

Although many studies available to find the diagnostic value of abnormal and equivocal mp-MRI findings, to the author’s knowledge, data on the outcome for negative MRI is scarce in the literature [27] hence, we have focused on this group, aiming that it could help with patient counselling as well as similar studies in the future.

There are a few limitations to this study. Firstly, all biopsies included in this study were trans-rectal due to low rates of trans-perineal biopsies in our institution. All the MRI scans in this study were performed using a 1.5 T scanner. Although these scanners have inferior image quality in diffusion-weighted imaging series of multi-parametric prostate MRI compared to 3 T scanners [28], there is no evidence to prove 3 T scanners produce better results compared to 1.5 T scanners [14]. Although the image-guided fusion prostate biopsy technique has a better prostate cancer detection rate [29] all these biopsies in this study have been performed with cognitive target only. However, targeted biopsies in the study group were very minimal as they had been carried out on a majority of normal MRIs rather than those with a specific suspicious area.

## Conclusion

With normal or equivocal pre-biopsy mp-MRI findings, 18.5% cancer was demonstrated in this study and 9.8% of them were clinically significant. Avoiding a TRUS guided prostate biopsy based on normal mp-MRI alone should carefully be decided. Suspicious prostate on DRE and high PSA density could be considered as supporting factors to proceed with biopsy in these cases. Patients must be clearly educated about these aspects if a decision is made to avoid a biopsy after a normal or equivocal MRI.
